# Activins and Follistatin in Chronic Hepatitis C and Its Treatment with Pegylated-Interferon-*α* Based Therapy

**DOI:** 10.1155/2015/287640

**Published:** 2015-04-19

**Authors:** Bassem Refaat, Ahmed Mohamed Ashshi, Adel Galal El-Shemi, Esam Azhar

**Affiliations:** ^1^Laboratory Medicine Department, Faculty of Applied Medical Sciences, Umm Al-Qura University, Al-'Abdiyah Campus, P. O. Box 7607, Makkah, Saudi Arabia; ^2^Department of Pharmacology, Faculty of Medicine, Assiut University, Assiut 6515, Egypt; ^3^Special Infectious Agents Unit, King Fahd Medical Research Center, King Abdulaziz University, P.O. Box 80216, Jeddah 21589, Saudi Arabia; ^4^Department of Medical Laboratory Technology, Faculty of Applied Medical Sciences, King Abdulaziz University, P.O. Box 80216, Jeddah 21589, Saudi Arabia

## Abstract

Pegylated-interferon-*α* based therapy for the treatment of chronic hepatitis C (CHC) is considered suboptimal as not all patients respond to the treatment and it is associated with several side effects that could lead to dose reduction and/or termination of therapy. The currently used markers to monitor the response to treatment are based on viral kinetics and their performance in the prediction of treatment outcome is moderate and does not combine accuracy and their values have several limitations. Hence, the development of new sensitive and specific predictor markers could provide a useful tool for the clinicians and healthcare providers, especially in the new era of interferon-free therapy, for the classification of patients according to their response to the standard therapy and only subscribing the novel directly acting antiviral drugs to those who are anticipated not to respond to the conventional therapy and/or have absolute contraindications for its use. The importance of activins and follistatin in the regulation of immune system, liver biology, and pathology has recently emerged. This review appraises the up-to-date knowledge regarding the role of activins and follistatin in liver biology and immune system and their role in the pathophysiology of CHC.

## 1. Introduction

At the present time, infection with hepatitis C virus (HCV) is a major health problem worldwide and it is the main cause of end stage liver diseases [1, 2]. Only 20%–30% of infected persons are expected to resolve following the acute phase without any therapeutic intervention, while the remaining 70%–80% develop chronic hepatitis C (CHC) within six months of infection [3]. CHC is the most predominant cause of liver cirrhosis, liver transplantation, and hepatocellular carcinoma (HCC) in developing countries [4, 5]. CHC is currently treated with a combination therapy consisting of a weekly injection of pegylated-interferon-*α*- (Peg-IFN-*α*-) 2a or 2b plus a daily weight-based dose of ribavirin (RBV) [6, 7].

The dose of the recommended treatment is dependent on the viral genotype, where a 24-week regimen is recommended for HCV genotypes 2 and 3, and a 48-week protocol is recommended for genotypes 1 and 4 [8]. However, this standard of care is considered suboptimal because of its long duration, inability to cure about half of the patients, and numerous potentially severe side effects that could result in dose reduction and/or premature termination of treatment [3]. Despite the recent development of new antiviral drugs [8], the estimated cost is 60,000–100,000 USD and, therefore, it is expected that Peg-INF-*α* and RBV may still have a role especially for those patients living in developing countries and for whom access to the new drugs is not definite [9–13].

The monitor of response to Peg-INF-*α* treatment is currently based on the viral kinetics during the course of therapy and the viral load is measured at specific time points to measure the performance of the drugs [8]. The base time point at which clinicians can form a decision regarding the potential response to treatment is at week 4 followed by week 12 posttreatment commencement [2]. Hence, there is a compelling need to develop new sensitive and specific prognostic markers that could predict the response to treatment prior to the start of therapy.

Activins and their binding proteins, follistatin, are secreted by the hepatocyte and regulate the immune system and their serum concentrations have been shown to increase during CHC [1]. Activins and follistatin have recently been suggested as potential sensitive and specific prognostic markers for the prediction of achieving sustained viral response at the end of Peg-INF-*α* based therapy [1, 2]. The present review summarizes the roles of activins and follistatin in the regulation of immune responses and their role in liver biology and pathology with specific emphasis on CHC.

## 2. Methods

“PubMed” and “EMBASE” were searched using the terms “hepatitis C virus,” “chronic hepatitis C,” “liver,” “prevalence,” “diagnosis,” “treatment,” “pegylated-interferon-*α*,” “ribavirin,” “liver fibrosis,” “liver cirrhosis,” “hepatocellular carcinoma,” “live cancer,” “immune response,” “predictive markers,” “diagnostic markers,” and “treatment response” in combination with “activin” or “follistatin” for studies published between 1987 and 2014. Publications in the past 6 years were mostly selected but commonly referenced, and important older publications were not excluded. The reference lists of articles identified by this search strategy were also searched and those judged as relevant were included. For a study to be included, it needed to be primarily focused on the expression of activins and their related molecules by the hepatocyte and immune cells, their roles in normal and abnormal liver, and their serological diagnostic/prognostic values in CHC. Studies that were solely epidemiological in nature were excluded.

### 2.1. Immune Response to HCV Infection

**Figure 1:**
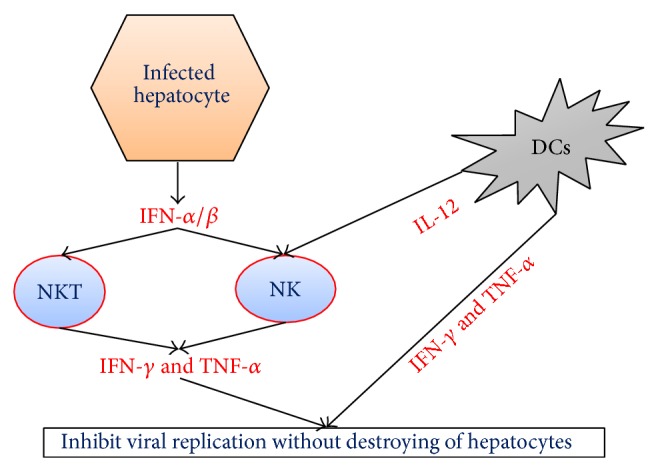
Summary of innate immune response against hepatitis C virus. Infected hepatocyte releases interferon type I (*α*/*β*) to activate natural killer (NK) and natural killer T (NKT) cells. Dendritic cells (DCs) also secrete interleukin- (IL-) 12 to activate NKT cells. Those cells later secrete interferon-*γ* (INF-*γ*) and tumour necrosis factor-*α* (TNF-*α*) to inhibit viral replication without causing destruction of the hepatocyte.

Infection with HCV leads to acute and chronic necroinflammatory liver disease [14, 15]. The immune system is not always able to control the infection and 70–80% of cases progress to chronic stage due to the escape of HCV from the immune system [15]. The release of interferon- (IFN-) *α* and interferon-*β*, which are also known as type 1 INF, is essential for the control of HCV during the acute phase [16, 17]. IFN-*α*/*β* activates a number of cellular genes, known as INF stimulated genes (ISGs), which prevent viral replication and spread to other liver cells (Figure 1) [18]. However, HCV is able to block type 1 IFN induction by the nonstructural proteins (NS3 and NS5A) and structural protein E2. HCV NS5A protein also induces the expression of interleukin- (IL-) 8 which is associated with the inhibition of IFN-*α* [15, 17].

Natural killer (NK) cells and natural killer T (NKT) cells are the first line of defense against HCV [19]. IFN-*α* and IFN-*β*, which are released by infected liver cells, are responsible for the activation of NK and NKT cells [20]. Furthermore, dendritic cells (DCs) release IL-12 that also activates the NK cells [20–22]. NK cells exert their antiviral activity through the production of IFN-*γ* and tumour necrosis factor-*α* (TNF-*α*), which inhibit the replication of the virus but without destroying liver cells (Figure 1) [23, 24]. In addition, they stimulate T helper 1 (Th1)/T cytotoxic (Tc) 1 responses [19, 25]. However, their role in controlling the infection is usually eliminated by HCV through blocking the production of IFN-*γ* via an interaction between HCV E2 protein and NK cell CD81 molecule [15, 17, 26, 27].

DCs also process and present viral antigens to specific immune system cells via class I and class II major histocompatibility complex molecules. Viral particles are captured by DCs through Toll-like receptors (TLRs) [28–30]. Activated DCs release a variety of cytokines including IL-12, TNF-*α*, IFN-*α*, and IL-10. These cytokines subsequently regulate and polarize the response of adjacent cells [29–32]. Mature DCs enter the lymph nodes after collection of viral epitopes to activate T cells in the specific immune system [18, 33].

The progression to chronic/adaptive response is initiated by CD4+-T cells, which provide help in activating cytotoxic and humoral responses. These cells can secrete Th1-cytokines including IFN-*γ*, leading to inflammatory response or Th2 cytokines (e.g., IL-4 and IL-10), which limit Th1 cytokine-mediated response and favour the development of humoral response [15, 34]. A multispecific, strong, sustained, CD4+-T-cell-specific Th1 response may be seen in infections with HCV progressing to resolution [17, 35]. However, when infection becomes chronic, a weak CD4-T-specific response with few specificities and scarce type 1 cytokine production is observed [17, 25, 29].

When specific immune response fails to control viral replication, the infected liver cells release chemokines resulting in the migration of nonspecific mononuclear cells into the liver, which are unable to control infection but lead to sustained liver damage [35–37]. Persistent inflammation also stimulates hepatic stellate cells, myofibroblasts, and fibroblasts, which lead to the development of liver fibrosis [17, 37]. The progression to chronic inflammation and the development of liver fibrosis is regulated by proinflammatory cytokines such as transforming growth factor- (TGF-) *β*, IL-6, and platelet-derived growth factor, among other stimuli [15, 38, 39].

### 2.2. Treatment of CHC

IFNs play an essential role in innate immunity by inhibiting the replication and spread of viral, bacterial, and parasitic pathogens [40]. They also modulate immune responses and exert cytotoxic, antitumoural, and antiproliferative effects in some cell types [41]. IFNs are commonly grouped into two types [18]. Type 1 comprises several subtypes, including IFN-*α*, IFN-*β*, IFN-*ω*, and IFN-*ε*, known as viral IFNs and they are produced from different cells such as white blood cells and fibroblast [17]. Type 2, which is known as IFN-*γ*, is induced by mitogenic or antigenic stimuli and is produced by T cells in response to an infection [18, 40].

INF-*α* was the first approved drug for treatment of patients with HCV [8]. The latter generation, Peg-INF-*α*, has a pegylated molecule (polyethylene glycol molecule) added to INF and it increases the half-life of the interferon molecule and allows for convenient dosing [42]. Two types of pegylated-INF, which differ in their pharmacokinetic and chemical properties, have been developed. The choice of Peg-INF-*α* based therapy was based upon the results of three randomized clinical trials that demonstrated the superiority of this combination treatment over standard INF-*α* and RBV [3, 6, 8, 43, 44] with a significantly improved sustained virological response (SVR) as compared with standard INF [8, 43].

HCV genotype is the most significant baseline predictor of response to therapy, and, therefore, the adjustment of HCV treatment, including the optimal duration and treatment protocol, is based on the genotype [3, 6]. Current guidelines recommend that all patients infected with HCV genotypes 2 or 3 should be treated with Peg-INF-*α* and low dose (800 mg) RBV for 24 weeks with an estimated SVR of about 80%. Coherently, patients with genotypes 1 and 4 could be treated with Peg-INF-*α* plus standard weight-based RBV (1200 mg) for 48 weeks with an estimated SVR of about 50% of cases [3, 6, 8, 43–47].

When no significant reduction in the viral load is seen after 3 months (12 weeks) of antiviral therapy for genotypes 1 and 4, patients are usually regarded as nonresponders and treatment is stopped. Treatment is also stopped when patients show a breakthrough, defined as a rise of the viral load during treatment after an initial decline. Relapses, with detection of the viral RNA in the blood despite initial negativity at the end of treatment, also occur mostly within 6 months following the termination of therapy [3, 6, 8, 43–47].

Almost all patients treated with Peg-INF-*α* and RBV experience one or more drug induced side effects during the course of therapy. One of the barriers to adherence in combination therapy for CHC is the incidence of treatment associated adverse events that can lead to dose reductions or sometimes premature discontinuation [3, 6, 8, 43–47]. In the registered trials of Peg-INF-*α*-2a and Peg-INF-*α*-2b plus RBV, 10% to 14% of patients had to discontinue therapy due to an adverse event [3]. The adverse effects include thyroiditis, flu-like syndrome, hematological disorders, and depression [3, 48–52].

### 2.3. Currently Used Predictors of Treatment Outcome

A significant proportion of patients infected with HCV still do not respond to Peg-IFN-*α* based therapy, especially those who are infected with genotypes 1 or 4 [3, 47, 53]. Therefore, predictive factors that identify potential nonresponders are needed to limit drug exposure in patients unlikely to benefit from treatment and to save healthcare resources [2].

Measuring the rate of viral clearance from serum is helpful in predicting the likelihood of a response to therapy, for determining the optimal duration of therapy and as a stopping rule for patients with CHC. Accordingly, there has been intense interest in tailoring treatment regimens for individual patients using viral kinetics. This approach may have the benefit of limiting exposure to Peg-INF-*α* and RBV, thus potentially leading to reduced toxicity and a cost savings [8, 11, 44, 46, 47].

Currently viral predictive factors are used to assess the response to treatment and they have shown a moderate precision in predicting the outcome of therapy [2, 3, 47]. Several types of virological responses may occur, labelled according to their timing relative to treatment [2, 3, 47]. The main goal of therapy in HCV infection is to achieve a SVR, currently defined as undetectable HCV-RNA in peripheral blood determined with the most sensitive PCR technique 24 weeks after the end of treatment. This goal is mainly regarded as a “virological cure” and it is equivalent to eradication of HCV infection and cure of the underlying HCV-induced liver disease [2, 3, 47].

There are several other virological responses that are currently used to predict the treatment outcome. CHC patients without an early virological response (EVR; HCV RNA either undetectable or decreased by > 2 log^10^ after 12 weeks following start of treatment) do not achieve a SVR with a negative predictive value of 97-98%. Thus, in the absence of an EVR, treatment should be stopped.

In patients with CHC treated with Peg-IFN-*α* based therapy, a rapid virological response (RVR, serum HCV RNA undetectable after 4 weeks after start of treatment) is usually used as an indicator of EVR at week 12 and can also predict SVR [3, 47]. Patients who become HCV-RNA negative after 4 weeks have the best chance of achieving a SVR [3, 47]. The rapidity of viral elimination may be a useful guide to tailoring how long treatment should be continued in patients with an RVR or EVR.

### 2.4. Potential New Predictors for Treatment Outcome

Substantial evidence has recently emerged to support the role of the host immune response in the outcome and pathogenesis of HCV infection [1, 3, 54]. Several studies have investigated potential host markers to assess the response to treatment [54, 55]. Although several molecules have been identified, the majority are expressed at the liver cell surface level and cannot be assessed in peripheral blood [54, 55]. Additionally, none of them combine accuracy, reproducibility, and simplicity in assessing treatment outcome and they have a low negative predictive value compared to viral predictive factors [54, 55]. Therefore, there is a compelling need to develop new serum markers and algorithms that provide a more sensitive and a specific tool for the assessment of treatment outcome in patients infected with chronic HCV and treated with Peg-INF-*α* based therapy [2].

Innate immune systems are important for the initial step of viral infection. Numerous studies have indicated that failure of the cellular immune response, including Th1 hyporesponsiveness, cytotoxic T lymphocyte exhaustion, excessive function of CD4+, CD25+, and FOXP3+ regulatory T cells, and failure of lymphoid cells via direct binding and/or infection in B cells, T cells, NK cells, and DCs, occurs in CHC patients [15, 56]. In HCV infection an inappropriate ratio of proinflammatory and anti-inflammatory cytokines may either determine different outcomes of the infection or affect the benefit of antiviral treatment [15, 31, 56].

INF-*α* activates a large number of ISGs including TLRs [57], TNF-*α* [58], and ILs [59]. IFN-*α* also enhances the activity of lymphocytes, macrophages, and NK cells and it activates neutrophils and monocytes [19, 25, 35, 60]. IFN-*α* alters the immune response in patients with CHC from Th-2 to a Th-1 mediated pattern [61]. Th-1 cytokines mediate response and favour the eradication of the virus [15, 35]. INF-*α* promotes Th-1 response through the increase in the production of IFN-*γ*, IL-2, and TNF-*α* by the hepatocyte and immune cells [18, 37]. IFN-*α* also inhibits the release of IL-6 and IL-10, which regulates Th-1/Th-2 cytokine balance, in patients with CHC [59, 62]. Additionally, IFN-*α* alters the production of immunoglobulin and decreases T-regulatory cell function [17, 63].

INF-*α* could also promote viral eradication through the modulation of TGF-*β* [38, 39, 64, 65]. TGF-*β*s are secreted by hepatic stellate cells, myofibroblasts, and fibroblasts during CHC and they can lead to the development of liver fibrosis [38, 39]. Gathered data from published reports suggest that activin-A and follistatin, which are members of the TGF-*β* superfamily, might be targets for Peg-INF-*α* based therapy for the eradication of HCV. Activins and follistatin have been described as major regulators of the liver biology and pathology and they are also major regulators of the immune system. Therefore, activins and follistatin have recently been proposed as potential sensitive and specific predictors of treatment outcome in chronic infection with HCV [1, 2].

### 2.5. Structure, Cell Signalling, and Regulation of Activins Bioactivities

Activins were originally isolated from porcine follicular fluid in 1986 and were named after their stimulatory effect on the secretion of follicle stimulating hormone from the pituitary gland [66, 67]. Activins and their binding protein, follistatin, were later found to be secreted by the hepatocyte and immune cells where they function as paracrine and autocrine factors to regulate a variety of liver functions [68–71] and immune responses [72, 73], respectively.

Activins are homo- or heterodimers of two *β*-subunits (*β*A and *β*B), and the different dimerization of subunits by a disulphide bond forms three mature proteins named activin-A (*β*A-*β*A), activin-B (*β*B-*β*B), and activin-AB (*β*A-*β*B) [67, 74]. The human activin *β*A-subunit and *β*B-subunit genes are located on chromosome 7 locus 7p14-p15 and chromosome 2qcen-q13, respectively [75]. The mRNA of both subunits encodes a preproprotein, their mature regions illustrate about 70% sequence homology, and both of them lack recognized glycosylation sites [76].

Activins mediate their actions by binding to a complex of transmembrane serine and threonine kinase receptors. These activin receptors are classified into type I and type II receptor groups, comprising the activins type IIA and type IIB receptors (ActRIIA and ActRIIB) [77]. Activins can bind their individual receptor type II when expressed alone but fail to bind to type I receptor in the absence of type II receptor [78]. However, both types I and II receptors are necessary to generate a high-affinity complex with the ligand, as well as for signalling [79].

The coordinated synthesis of follistatin with activin is the main regulator of the local bioactivity of activin since binding of activin to follistatin is almost irreversible [77]. The activin-follistatin complex generally consists of one activin dimer and two follistatin molecules [80]. In general, activin-A, activin-AB, and activin-B bind to follistatin with similar affinity [81].

Both activin subunits, activin type II receptors, and follistatin were previously localised within the hepatocyte of several species including human and pathological alterations in their hepatic and serum concentrations have been reported in several hepatic pathologies suggesting a role for these proteins in the regulation of the physiological functions and the pathogenesis of liver [82, 83]. Therefore, these proteins have been proposed as potential sensitive and specific markers to monitor the progress and outcome of CHC and its treatment with Peg-INF-*α* based therapy [2].

### 2.6. Activins in the Regulation of Immune System

**Figure 2:**
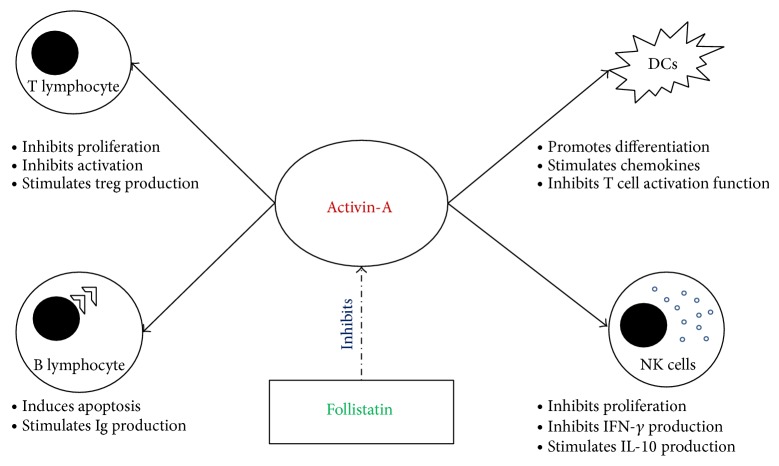
The role(s) of activin-A in the regulation of major immune cells involved in the immune response against hepatitis C virus. The actions of activin-A are inhibited by follistatin.

Activin-A is a pleiotropic cytokine that participates in developmental, inflammatory, and tissue repair processes. Activin-A expression has been detected in many immune cells [84, 85] including DCs [86, 87], T and B lymphocytes [73, 88, 89], and NK cells [87] (Figure 2).

Similar to TGF-*β*, activin-A could act either as a pro- or anti-inflammatory molecule depending on the type of a disease and the cellular and immune contexts [85, 90]. Activin-A has been found to regulate both innate and humoral immunity processes and the production of several cytokines [85, 90, 91]. It is rapidly induced during systemic inflammation, inhibits the acute phase reaction, and can antagonize IL-6 effects [92, 93]. Activin-A and follistatin signals are also involved in tissue repair and fibrosis in many organs including skin, lung, pancreas, kidney, and liver [94, 95].

The effects of activin-A on T helper cell–mediated immunity are elusive and critical for allergic and autoimmune diseases [73, 96]. The production of activin-A is increased in activated CD4+ T cells at the mRNA level and its secretion is significantly upregulated in activated Th-2 clones compared with Th-1 clones [88, 91]. Furthermore, activin-A regulates lymphocyte functions by inhibiting proliferation and differentiation into effector cells, and it also induces apoptosis in B and T cells [89, 97, 98].

It also appears that activin-A exerts a complex range of immunoregulatory functions, encompassing effects on the proinflammatory functions of activated monocyte/macrophages; development and maturation of monocyte-derived DC; recruitment, development, and/or survival of T cells, B cells, NK cells, and mast cells; deviation of local immune responses toward a type 2 phenotype; and production of antigen-specific regulatory T cell subsets [99]. Activin-A production is also increased in activated CD4+ T cells at the mRNA level and its secretion is significantly upregulated in activated Th-2 clones compared with Th1 clones [88].

Several studies have provided evidence that endogenously produced activin-A suppresses antigen-specific Th-2 responses and protects against airway hyperresponsiveness and allergic airway disease in mice. Activin-A also suppresses Th1-driven responses, pointing to a broader immunoregulatory function. Additionally, blocking the actions of IL-10 and TGF-*β*1 reverses activin-A-induced suppression on T helper cells [100–102].

Follistatin, on the other hand, is upregulated during inflammation and it appears that it is driven not only by activin itself [90, 103–105] but also by other cytokines, particularly, IL-1*β*, TNF-*α*, and IFN-*γ* [99, 106, 107]. The observation that expression of follistatin is altered in tissues during fibrosis also suggests a role for endogenous follistatin in controlling the fibrotic effects of activin [95, 108–110].

### 2.7. Activins in Liver Biology and Pathology

#### 2.7.1. Activins and Follistatin in Normal Liver

Activin-A is expressed in hepatocytes and has been shown to be a negative regulator of hepatocyte cell growth [82, 83]. The expression of activin-A is relatively low in normal liver [68]. Activin-A is also involved in the maintenance of constant liver mass and the administration of follistatin leads to hepatocyte proliferation [111–113]. Treatment of a human hepatoma cell line with activin-A antisense oligonucleotides, in contrast, stimulated cell proliferation suggesting a growth inhibitory function of endogenous activin-A [114]. Follistatin administration by intraportal infusion or adenovirus-mediated overexpression also caused DNA synthesis and liver growth in normal rat livers [82, 115].

A balance between cell proliferation and apoptosis is crucial for regulating normal liver function [82, 83], since abnormalities in liver regeneration may contribute to chronic hepatitis, cirrhosis, and cancer [1]. Cellular control of activin expression appears to occur at many levels, including the amount of growth factor, dimer combinations formed, the presence of binding proteins, receptor recruitment, and presence of other peptides [116]. Activin-A is upregulated during the development of liver cirrhosis concomitant with an increase in hepatocyte apoptosis [117, 118]. Activin *β*C-subunit mRNA appears to be predominately expressed in the liver [117, 118] and is downregulated 12 h after partial hepatectomy in the rat, suggesting that it also may be a negative regulator of liver cell growth [119]. The presence of activin subunits, follistatin, and activin receptor mRNAs and the ability of these proteins to affect hepatic cell division imply that these growth factors may play a key role in the process of liver regeneration [82, 83].

#### 2.7.2. Activins and Follistatin in Hepatic Disorders

**Figure 3:**
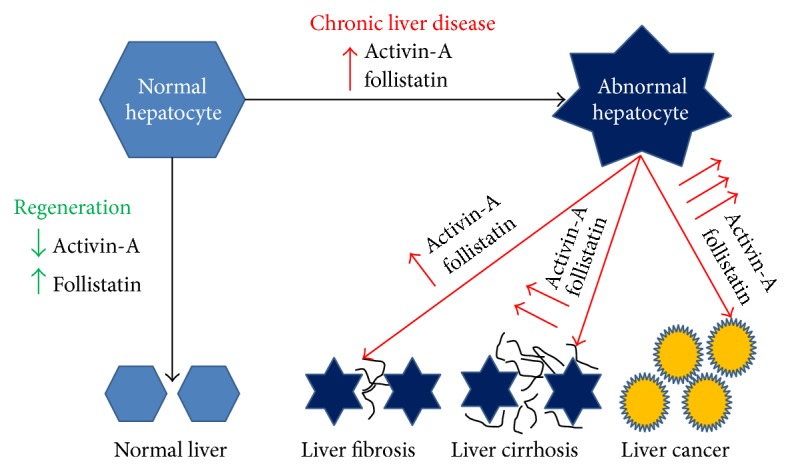
The production of activin-A and follistatin by normal and abnormal hepatocyte. Activin-A and follistatin regulate hepatocyte regeneration in healthy liver. Pathological increase of both activin-A and follistatin by the hepatocyte is associated with several liver diseases including fibrosis, cirrhosis, and hepatocellular carcinoma.

Liver inflammation and fibrosis are closely linked and a number of observations indicate an important role for activins in these processes (Figure 3). Activins *β*A-, *β*C-, and *β*E-subunit expression levels were found to increase during carbon tetrachloride (CCl4) induced fibrosis in rat livers and have been implicated in destruction of hepatocytes and elevated extracellular matrix production [82, 83, 120].

The expressions of activin subunits and follistatin have been reported in the liver of several species and alteration in their expression has been linked with a variety of liver diseases [82]. Experimental studies have shown that activin-A has a range of effects on hepatic cells that may support a pathogenic role for this cytokine in hepatic disorders [1, 82]. These include induction of hepatocyte apoptosis and inhibition of hepatocyte growth [83, 121].

Prolonged and/or significantly increased expression of activin-A is involved in the development of fibrosis. The expression of activin-A was increased in liver fibrosis in rat and it was mainly localised around the fibrotic areas [122]. Activin *β*A-subunit expression was also upregulated during CCl4 induced fibrosis in rat livers and has been implicated in the induction of hepatocyte apoptosis and production of extracellular matrix [83, 118, 123–125]. The production of *β*A-subunit also increased in rat liver fibrosis and cirrhosis induced by injection of dimethylnitrosamine [82, 122]. Furthermore, the promotion of extracellular matrix production by activin-A in hepatic stellate cells and tubulogenesis of sinusoidal endothelial cells indicates that the latter contributes to the process of tissue architecture restoration during liver regeneration [121].

Other studies have also suggested that follistatin is overexpressed in the majority of chemically induced rat liver tumours [126]. Administration of follistatin to normal and preneoplastic rat hepatocytes in primary culture stimulated whereas recombinant activin-A inhibited DNA synthesis rates predominantly in the preneoplastic hepatocytes [126], suggesting that deregulation of the balance between activins and their antagonist may contribute to the growth advantage of preneoplastic hepatocytes [82].

Coherently, clinical studies also suggest the involvement of activin-A in the pathogenesis of a range of liver disorders [1, 2]. Serum levels of activin-A are increased in patients with chronic viral hepatitis and alcohol-induced cirrhosis [1, 2, 127–129], as well as in patients with acute liver failure [130, 131]. Serum activin-A significantly increases in liver fibrosis and cirrhosis induced by viral and nonviral factors [129]. Serum levels of activin-A and follistatin were found to be increased in patients with acute liver failure due to paracetamol overdose and non-A to E hepatitis and it was suggested that increased production of activin-A could be a contributing factor to the impaired liver regeneration [131]. Lin et al. (2006) have also suggested that a decreased follistatin/activin-A ratio in the blood may be an indicator for the severity of liver injury in hepatitis-related acute liver disease [130]. Additionally, increased serum levels of both activin-A and follistatin have been found in the circulation of patients suffering from liver cirrhosis and HCC [127, 132, 133].

Serum activin-A was linked to viral replication in chronic hepatitis B and hepatitis C [128], and it correlated significantly with liver damage associated with HCV [127]. Hence, it has been suggested that pathological alteration in the hepatic expression of activin-A and follistatin could lead to impaired liver regeneration [131] and the development of liver fibrosis, cirrhosis, and hepatocellular carcinoma [134].

### 2.8. Activins and Follistatin in CHC

Currently, the published studies on activins and follistatin in CHC are few (Table 1) and the available knowledge is limited [1]. The first study was reported among Australian patients by Patella et al. [128] and demonstrated a significant increase in the serum levels of activin-A with nonsignificant change in follistatin in archived samples of 47 CHC patients. Additionally, there was no significant correlation between these proteins and liver enzymes [128]. Meanwhile, the alliterating effect of CHC on serum activin-A was further observed among Egyptian CHC patients by Elsammak et al. (2006), who did not include follistatin, and the authors have demonstrated a significant positive correlation between activin-A and the Child-Pugh scores in 30 patients [127]. By contrast and more recently, Voumvouraki et al. (2012) did not detect significant changes in serum activin-A in 18 Greek patients with CHC [129].

Recently, we have reported two studies conducted on Saudi patients infected with CHC genotypes 1 and 4 and the results have shown a significant increase in serum activin-A and activin-B and a significant decrease in follistatin in those patients [1, 2]. Furthermore, serum concentrations of activin-A, but not activin-B and follistatin, correlated significantly with AST platelet ratio index, serum albumin, and the viral load. Consistently, we have also demonstrated that Peg-INF-*α* based therapy modulated the concentrations of both serum activin-A and follistatin and their levels were comparable to control in those patients who responded to treatment and achieved a sustained viral response [2].

Hence, we have suggested that both proteins could be involved in the immune response to HCV and they could represent potential targets for Peg-INF-*α* based therapy during the treatment of CHC. We also propose that serum activin-A, follistatin, and activin-A/follistatin ratio index could be potential sensitive and specifics markers for the prediction of treatment response in CHC prior to the commencement of Peg-INF-*α* based therapy [2].

The inconsistent outcomes reported by the aforementioned studies on activins and CHC could be related to the types of viral genotype included in these studies. Genotypes 1 and 4 are known to be more aggressive and harder to treat and are associated with more severe form of the disease compared with genotypes 2 and 3 [135]. None of the early 3 studies by Patella et al. [128], Elsammak et al. [127], and Voumvouraki et al. [129] reported the viral genotype(s) in their study populations (Table 1). Therefore, the variation between those studies in the correlation of activin-A with viral load and liver damage despite the observed significant increase in serum activin-A in CHC could be due to differences in the viral genotypes since the most prevalent genotypes in Australia are genotypes 1 (54%) and 3 (37%) [136], while in Saudi Arabia and Egypt genotypes 4 (69%) and 1 (25%) are most frequent [4, 137].

Furthermore, the discrepancies in the results of activin-A reported from Greece with the other reports, including ours, regarding activin-A could either be due to their small sample size (18 patients) and/or differences in viral genotype as the most predominant genotypes in Greece are 1 and 3 (40% each) [138, 139]. Hence, the inclusion of the different genotypes and the other activin mature dimeric proteins (e.g., activin-AB) in future studies is still required to demonstrate the clinical value(s) of activins and follistatin in CHC and the prediction of Peg-INF-*α* based therapy.

### 2.9. How Could Activin Influence the Development of Chronicity and Liver Damage in HCV Infection and Affect Its Treatment Outcome with Peg-INF-*α* Based Therapy?

As mentioned earlier, the immune response to HCV and the modulation of the immune system by Peg-INF-*α* based therapy towards a Th1 pattern are the main mechanisms for the prevention of viral replication and induction of viral eradication [15, 17, 19, 20, 25]. Currently, there is no direct evidence in the literature on the role(s) of activins and its binding protein in the immune response to HCV and the apparent gaps in our knowledge are mainly due to the limited number of studies that directly investigated activins and their related proteins in CHC. However, there are phenomena, which are becoming increasingly clear, that can suggest several possible pathways and signals by which activins could affect HCV replication, chronicity, and severity of its associated liver damage. Furthermore, gathered data from published reports suggest that activin-A/follistatin might be a target for Peg-INF-*α* based therapy for the eradication of HCV.

Beside their established roles in the regulation of cell growth, differentiation, and organogenesis, recently emerged data indidates the importnace of activins in the regulation of the immune system in viral and nonviral infections [99, 140]. Furthermore, activins modulate the functions of several key immune cells (e.g., NK cells and DCs) and the expression of several cytokines including IL-6, IL-8, IL-10, and TNF-*α* [84–87], which are known to play a crucial role in CHC and in the eradication of the virus by Peg-INF-*α* based therapy [18–20, 23, 24]. The potential mechanisms relating activins to CHC and its treatment with Peg-INF-*α* based therapy are discussed below in detail.

#### 2.9.1. Activins/Natural Killer Cell Hypothesis

NK cells constitute first line of defense against viral infections [18, 19]. They rapidly recognize and lyse virus-infected cells, inhibit viral replication, and exert immune regulatory functions. NK cells constitute approximately 30% of resident lymphocytes in a normal liver and may account for as many as 60% of lymphocytes in HCV infection [141]. NK cells also play an important role in the regulation of both innate immunity and adaptive immunity via direct interaction with DCs in the lymph node or within inflamed tissues via production of IFN-*γ* [142]. It has been reported that NK cells can inhibit HCV replication* in vitro* by both IFN-*γ* mediated noncytolytic as well as granzyme/perforin and TRAIL-mediated cytotoxic mechanisms [143].

Werner et al. (2014) have illustrated that NK cell cytotoxicity and their production of antiviral IFN-*γ* are increased with Peg-IFN-*α*/ribavirin combination therapy compared to Peg-IFN-*α* monotherapy [144]. In this regard, recent evidences have suggested that selective impairment of NK cell activity is related to the establishment of CHC and viral persistence [145]. The cytotoxic functions of NK cells and their production of IFN-*γ* to inhibit viral replication and prime the adaptive immune response are much lower during chronic HCV compared with acute HCV infection [32, 145–147]. More importantly, other studies have also provided evidence that this impairment of NK cell cytotoxic function is more pronounced in intrahepatic NK (IH-NK) than the peripheral NK cells in patients with CHC [148]. One possible explanation for this selective dysfunction could be attributed to the overexpression of IH-NK cell inhibitory receptors, which should be taken into consideration for novel therapeutic strategies that intend to involve direct activation of IH-NK cells or to block IH-NK cell inhibitory receptors in order to enhance the induction of antiviral cytotoxic activities [145, 149].

Activins are known suppressor of NK cell functions and activin-A has attenuating effects on several human NK cell functions similar to TGF-*β* [65, 150]. Activin receptors are expressed on NK-cells and activins could directly regulate the functions of NK cells by [150] (1) downregulating the T-box transcription factor and IFN-*γ* mRNA; (2) suppressing NK cell IFN-*γ* production as potently as TGF-*β*; and (3) suppressing NK-cell CD25 expression, proliferation, and sculpting NK-cell cytokine and chemokine profiles. The ability of activin-A to suppress NK cell function and IFN-*γ* production may be critical in preventing inappropriate immune cell activation and tissue damage and highlighting the potential of antagonizing activin-A signalling* in vivo* to enhance NK cell-mediated immune functions and adaptive immunity [150]. In addition, Seeger et al. (2014) have confirmed the modulatory effects of activin-A on NK-DC cells functional interactions and their production of certain cytokines and chemokines [87]. Taken together, it could be speculated that the observed overexpression of activin-A during the course of HCV infection could, at least in part, contribute to NK cell dysfunction and viral persistence and subsequently the development of CHC and/or failure of Peg-INF-*α* based therapy. However, further studies are required to illustrate the possible role of activins in the regulation of NK cells during CHC infection.

#### 2.9.2. Activins/Cytokines Hypothesis

The majority of cases infected with HCV develop chronicity mainly due the viral modulation of the actions of endogenous INF-*α* and the alteration in the production of several pro- and anti-inflammatory cytokines at the hepatic and systemic levels [15]. These patients are at greater risk of developing significant liver damage including fibrosis, cirrhosis, and liver cancer [17, 25]. Now there is compelling evidence that there are various proinflammatory cytokines such as TNF-*α*, IL-6, IL-1*β*, and IL-8 that are significantly implicated in the progression of chronic HCV infection and its mediated liver damage and fibrosis [1, 15, 17, 151].

Activin-A, as a component of the innate immune response, can modulate the adaptive immune response and the release of several key cytokines [90]. For example, in acute inflammation caused by a lipopolysaccharide challenge, serum activin-A levels were increased long before those of TNF-*α*, IL-1*β*, and IL-6, and blocking activin bioactivity by follistatin had resulted in a significant decrease in the serum levels of these cytokines [92]. The authors have therefore suggested that activin-A is synthesised and stored in the immune cells and its early release is essential for the modulation and production of the proinflammatory cytokines* in vivo* and the effects of activin on these key cytokines may occur by cross-talk between the cytokine producing cells and activin signalling pathways [90, 92].

Recently, Rocha et al. (2012) have found that activin-A stimulates the release of IL-8 in patients with endometriosis [152]. These recent findings suggest that the increased levels of activin-A during CHC could also contribute in the progression towards chronicity by modulating the expression of IL-8 which is known to be also induced by HCV NS3 and NS5A and structural protein E2 to inhibit the actions of IFN-*α* [15, 17]. Moreover, it has been shown that activin-A has a direct influence on IL-6 production in critically ill patients infected with influenza A virus (H1N1) [153]. Coherently, we have recently reported that the serum levels of both activin-A and activin-B, IL-6, and TNF-*α* were significantly increased, while serum follistatin was significantly decreased, in patients infected with HCV genotypes 1 and 4, and activin-A showed the strongest positive correlation with the viral load, liver fibrosis, IL-6, and TNF-*α*. Hence, we hypothesized that dysregulation of activins/follistatin axis may be associated with enhanced viral replication and liver injury in chronic HCV infection [1].

Additionally, Peg-INF-*α* increases the production of TNF-*α* [58] and serum follistatin [2] and decreases serum IL-6 and serum IL-10 [62] and activin-A [2] in patients with CHC. Moreover, activin-A has been reported to modulate the release of INF-*γ* [102, 154], which plays an important role in controlling CHC following Peg-INF-*α* based therapy [155]. Hence, the observed restoration of the pathological alteration in activin-follistatin axis follwoing Peg-INF-*α* based therapy suggests that activins and their binding protein play a signifcant role in the progression to chronicity, development of liver fibrosis during HCV infection, and response to INF therapy by modulating the release of several proinflammatory cytokines. However, further studies are needed to demonstrate the effect(s) of activins and follistatin on the production of other well established panel of cytokines known to be involved in the development of CHC (e.g., IL-10, IP-10) and whehter Peg-INF-*α* based therapy has a direct effect on the concentrations of serum activin-A and follistatin during the treatment of CHC.

#### 2.9.3. Activins/Toll-Like Receptors Hypothesis

Toll-like receptors (TLRs) are pattern recognition receptors that play an important role in host defense by recognizing pathogen-associated molecular patterns. TLRs sense pathogen-associated molecule patterns and activate antiviral mechanisms including the intracellular antiviral pathways and the production of antiviral effectors like IFNs [156]. Recent studies indicate that impairment and/or polymorphisms of TLR signalling play an important role in progression of chronic HCV and HBV infections [157, 158]. Being a persistently infecting virus, HCV developed sophisticated escape strategies from both the innate and adaptive immune systems, and experimental data suggest that impaired activation or functions of TLRs contribute to HCV chronicity and treatment failure [159].

Other studies have also suggested that HCV utilizes its NS3/5A serine protease to facilitate chronic infection as it induces specific proteolysis to inhibit TLRs signalling. Thus, it has recently been suggested, and is currently under development, that enhancement of TRLs activity (e.g., by TLR-ligand based therapies) may represent a promising approach in the treatment of viral hepatitis including chronic HCV [160].

As activin-A has shown inhibitory effects on TLRs, particularly on TLR-4 [161, 162], it can be hypothesized that the reported increase in the expression of activins during CHC, in part, contribute in HCV chronicity and its escaping from the innate immune system by modulating the expression of TLRs. More studies are still needed to measure the role(s) of activins and follistatin on TLRs during the course of HCV infection.

### 2.10. Activins in the Era of the New Direct-Acting Antiviral Drugs for the Treatment of CHC

The recent breakthrough in the treatment of CHC and the introduction of the new directly acting antiviral agents is promising and the results of several clinical trials have shown higher response rates (about 95%) and shorter duration of treatment compared with the traditional therapy [163–165]. These agents are NS3A and NS5A inhibitors that prevent the viral defense mechanisms initiated by these nonstructural proteins to inhibit the effects of endogenous INF-*α*. This has led to the expectation of possible INF-free regimen for the treatment of CHC with higher success rates and avoidance of the associated complications with the traditional treatment [163–165].

However, it could be argued that this new therapy is still, by one way or another, dependant on the actions of INF-*α*, which is produced endogenously by the host immune cells to control the viral infection. Additionally, the new therapy is still currently used in combination with the traditional treatment except in those cases with contraindications for the use of Peg-INF-*α* [164]. Hence, understanding the relation between endogenously produced INFs and activin-follistatin axis could provide new insight into the treatment of CHC and/or the prevention of the associated hepatic and extrahepatic complications. Even with the official announcement of the INF-free era, the cost of the new drugs is high and most probably many patients are not expected to be able to afford the cost of the treatment, which could pose a financial burden on them and their families. Hence, the traditional therapies with Peg-INF-*α* may still have a role in the treatment of CHC [2, 166].

Serum activins and follistatin could have a role in the future if proven to be a sensitive and specific predictive tool for treatment outcomes using Peg-INF-*α* based therapy. Their serum levels could thus provide a useful clinical mean for the identification of those who could not respond to the traditional therapy and hence a better direction and management of the healthcare resources, especially in those poor countries where the governments can only support the new treatment for selective cases [2, 166].

## 3. Conclusions

Chronic hepatitis C is a major worldwide health problem affecting about 3% of the world population. Additionally, about 3-4 million persons are acquiring new infection each year. Untreated cases of CHC can result in liver fibrosis, cirrhosis, liver failure, and development of liver cancer. The traditional therapy of CHC is the combination of Peg-INF-*α* with ribavirin and the duration of the treatment is based on the viral genotype. G1 and G4 are the least responsive genotypes to the current treatment and cure is only achieved in about 50% of the cases while G2 and G3 are associated with 80% of achieving SVR.

Although new directly acting antiviral drugs have been developed, the treatment of CHC is still based on a weekly injection of Peg-INF-*α*-2a or Peg-INF-*α*-2b plus a daily weight-based dose of ribavirin with or without the new antiviral therapy depending on the progression of liver damage and the presence of other extrahepatic manifestations. Furthermore, the new antiviral drugs are expensive and, therefore, Peg-INF-*α* based therapy could still be the standard of care especially for treatment of naïve patients with no liver cirrhosis and/or for those living in developing countries and for whom access to the new drugs is not definite due to the high cost.

Activins and their binding protein, follistatin, are expressed by the hepatocyte and have been described as major regulators of liver biology, liver regeneration, and liver pathology. Additionally, they play an important role in the regulation of the immune system and the pathogenesis of inflammatory and fibrotic human diseases. Significant dysregulation in serum activin-A/follistatin axis also occurs during liver diseases and, therefore, could be utilized as diagnostic/prognostic biomarkers for liver injury.

The currently used markers for the prediction of treatment success of Peg-INF-*α* are based on viral kinetics, which do not combine accuracy, reproducibility, and simplicity in the prediction of treatment outcome. Hence, there is a compelling need to develop new markers and algorithms that provide a more sensitive and specific tool for the prediction of outcome during the treatment of CHC with Peg-INF-*α*. The introduction of such predictive tool(s) would offer the classification of patients according to their response to Peg-INF-*α* based therapy and could subsequently provide a more cost-effective approach for subscribing the new antiviral therapy only for those who are not expected to respond to the traditional therapy or have contraindications for the use of INF based therapy.

Abnormal levels of serum activin-A and follistatin have been documented in several liver pathologies including viral hepatitis C. Additionally, CHC and Peg-INF-*α* based therapy have been recently shown to modulate the serum concentrations of both proteins. Hence, serum activin-A and follistatin could provide potential novel sensitive and specific noninvasive markers for the prediction of response to Peg-INF-*α* based therapy during the treatment of CHC. Despite the fact that activins and follistatin are involved in the regulation of many of the immune cells and cytokines that are involved in the immune response to HCV, currently there is lack of studies on the role(s) of these molecules during the course of infection with HCV. Hence, further studies are needed to elucidate the clinical value(s) of activins and their related molecules in the pathogenesis of CHC, prediction of the treatment outcome during the use of Peg-INF-*α* based therapy, and the effect(s) of the new antiviral agents on these immune regulatory proteins.

## Figures and Tables

**Table 1:** Characteristics of published studies that investigated the diagnostic value of serum activin-A ± follistatin in patients with chronic hepatitis C (CHC).

Research group	Publication year	Study design	Number of patients	HCV genotype	Serum target markers	Main findings	Reference number
Patella et al.	2001	Retrospective	15 normal control 22 CHB 47 CHC	Not reported	Activin-A Follistatin	Significant increase in serum activin-A in viral hepatitis (B and C) but not serum follistatin No correlation between serum activin-A and follistatin with viral load and liver enzymes	[128]

ElSammak et al.	2006	Prospective case-control	30 normal control 30 Hepatitis C 30 CHC + SHF 30 CHC + HCC	Not reported	Activin-A	Serum activin-A increased significantly in the study groups compared to control and it was the highest in HCC Serum activin-A correlated positively with the Child-Pugh scoring in the study groups	[127]

Voumvouraki et al.	2012	Prospective case-control	19 normal control 47 primary biliary cirrhosis 22 alcoholic cirrhosis 16 alcoholic fatty liver 18 CHC 20 CHC + cirrhosis 39 HCC	Not reported	Activin-A	No significant difference for serum activin-A in patients with activin CHC compared with control either in peripheral of portal vein samples There was no correlation between the cirrhosis stage and activin-A levels	[129]

Refaat et al. (a)	2014	Prospective case-control	40 normal control 40 CHC	Genotypes 1 and 4	Activin-A Activin-B Follistatin	Serum activin-A and activin-B significantly increased, while serum follistatin significantly decreased in CHC It was positive for activin-A and activin-B with viral load, APRI, IL-6, and TNF-*α* and negative with albumin No correlation was detected for follistatin	[1]

Refaat et al. (b)	2014	Prospective cross-sectional	40 normal control 33 CHC + no treatment 19 CHC at week 4 of therapy 22 CHC at week 12 of therapy 19 CHC at week 24 of therapy 21 responder to therapy 11 nonresponder to therapy	Genotypes 1 and 4	Activin-A Activin-B Follistatin	Serum activin-A and activin-B significantly increased, while serum follistatin significantly decreased in CHC Activin-A and follistatin, but not activin-B, at weeks 4, 12, and 24 after treatment initiation were similar to normal control Activin-A correlated positively and significantly with the viral load and APRI	[2]

CHB: chronic hepatitis B; SHF: schistosomal hepatic fibrosis; HCC: hepatocellular carcinoma; APRI: AST platelet ratio index.
